# Building a Comprehensive AED Program for K–12

**DOI:** 10.1177/1942602X251415545

**Published:** 2026-01-30

**Authors:** Karen Leal, Josie Tombrella

**Affiliations:** Doctor of Nursing Practice-Student, University of Texas Medical Branch School of Nursing, Galveston, TX, USA; Doctor of Nursing Practice-Assistant Professor, University of Texas Medical Branch School of Nursing, Galveston, TX, USA

**Keywords:** emergency care plan, emergency action plan, quality improvement, evidence-based best practice, advocacy, leadership, multidisciplinary collaboration, education, school nurse

## Abstract

Comprehensive automated external defibrillator (AED) programs with developed medical emergency response plans can improve sudden cardiac arrest outcomes in school-age children. This article explores literature on existing school AED program recommendations and the school nurse’s advocacy role in AED program implementation. This article also discusses three major themes identified: (a) The school nurse should advocate for school health and lead the school through implementation of a comprehensive AED program, (b) school campuses should implement an AED program, develop a cardiac emergency response plan (CERP), and practice mock drills, and (c) minimal AED program requirements should be researched, outlined, and written into law to promote safety on all school campuses. A collaborative approach to building a CERP, addressing barriers to implementation, and current legislation are discussed. By initiating changes within their role and building a comprehensive AED program, the school nurse can positively influence sudden cardiac arrest and cardiac emergency outcomes on their school campus.

On August 14, 2024, Landon Payton, a 14-year-old eighth grader in Houston Independent School District (ISD), died after receiving inadequate emergency care while experiencing a medical emergency in physical education during a routine day at school (Lehrer-Small, 2024). Every 3 days a young athlete dies of a sudden cardiac arrest (SCA), making SCA the leading cause of death for exercising young athletes in the United States (The Sports Institute, 2021). According to Project ADAM (automated defibrillators in Adam’s memory, 2024), defibrillation within 4 min of SCA provides the best odds of survival, which decreases up to 10% every additional minute. For school-age children and young athletes, quick access to an automated external defibrillator (AED) and a cardiopulmonary resuscitation (CPR) trained person can save the life of a child and greatly improve the chances of preventing severe disability (The Sports Institute, 2021).

According to Texas Children’s Hospital (2024), the national initiative Project ADAM began in 1999, after a series of young athletes’ sudden deaths, to encourage all schools in the United States to implement a heart safe program. With large hospital systems supporting a nationwide initiative, why are many K–12 school campuses still lacking a heart safe program more than 25 years later? This article explores considerations for the school nurse’s role in implementing emergency protocols on school campuses as well as potential barriers to achieving, implementing, and maintaining medical emergency preparedness.

## The Role of the School Nurse

Registered nurses primarily lead healthcare in the K–12 school setting and continuously collaborate with administration, staff, families, physicians, students, and school liaison officers. School nurse responsibilities generally include responding to all medical emergencies on campus, developing emergency action plans for at-risk students and sharing them with staff, performing annual emergency preparedness training for campus staff, and coordinating campus emergency medical preparedness. This centralized nursing position provides opportunities for school nurses to greatly impact health outcomes in the school population and the surrounding community. School nurse-driven initiatives to develop and maintain protocols for a comprehensive AED program at K–12 schools can improve the survival rate and recovery potential for students and young athletes suffering a SCA on school property. Survival rates after SCA more than double when CPR and AED use are initiated rapidly, emphasizing the critical role of a trained emergency response team and an established cardiac emergency response plan (CERP; American Heart Association, 2023).

**Figure 1.**
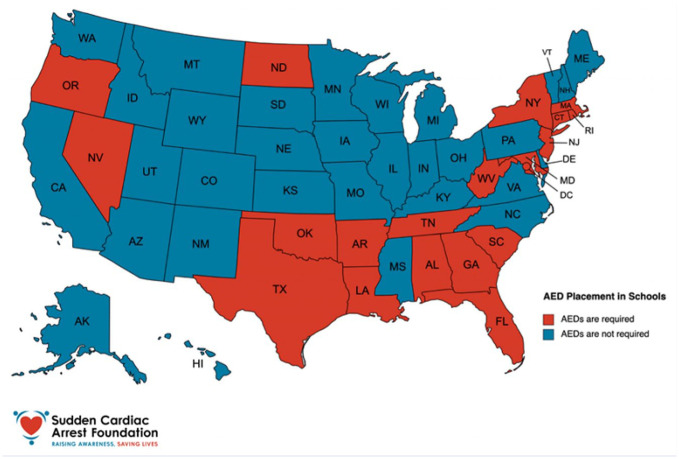
States With AED Requirements in Schools *Source*. Sudden Cardiac Arrest Foundation (n.d.). AED = automated external defibrillator.

With proactive emergency preparedness, the number one leading cause of death in exercising young athletes in the United States is not only survivable but also survivable with good outcomes. After investigation of the student’s death, on September 6, 2024, the Houston Chronicle reported 170 nonworking AEDs in Houston ISD schools, inadequately trained school staff in CPR and AED use, and a districtwide school nurse shortage (Partain, 2024). For Landon Payton, a school heart safe program might have saved his life. Across the United States, less than half of the states require AEDs to be present in schools or during sporting and extracurricular activities (Sudden Cardiac Arrest Foundation, n.d.). see Figure 1. While Texas does have laws in place, the tragic death of Landon Payton shows that having a law in place is only one component of a successful program. To ensure a school or district’s readiness for this type of emergency, school nurses should know best practices for supporting students who may experience cardiac arrest, as well as how to address barriers to implementing a comprehensive AED program.

## Best Practice Related to Sudden Cardiac Death in Schools

A literature review to determine best practices related to sudden cardiac death in schools revealed three major themes:

1. The school nurse should advocate for school health and lead the school through implementation of a comprehensive AED program (Boudreaux & Broussard, 2020; Nishiuchi et al., 2018; Saberian et al., 2018).
2. School campuses should implement an AED program, develop a CERP, and practice mock drills (Boudreaux & Broussard, 2020; Drezner et al., 2019; Kiyohara et al., 2019; Nishiuchi et al., 2018; Saberian et al., 2018).
3. Minimal AED program requirements should be researched, outlined, and written into law to promote safety on all school campuses (Boudreaux & Broussard, 2020; Drezner et al., 2019; Kiyohara et al., 2019; Nishiuchi et al., 2018; Saberian et al., 2018).

While instituting new laws requires time and advocacy and would help ensure that comprehensive AED programs are in place, the other two themes can be immediately addressed.

## School Nurses Can Lead the Way

For successful implementation and productive longevity, the campus comprehensive AED program must have an involved, active campus leader (Boudreaux & Broussard, 2020; Nishiuchi et al., 2018). On school campuses and within school districts, the school nurse leads health services, influences decision-making, advocates for programs that reduce risk, coordinates implementation of emergency plans, and provides direct care for emergent health care needs (National Association of School Nurses [NASN], 2024). As the school health leader, the nurse should be aware of state laws and school board policies regarding AEDs, staff CPR, and AED training. Existing school and district emergency medical protocols and guidelines should be reviewed. If none exists, the school or district emergency response team should be consulted to establish a policy. A campus cardiac emergency response plan should be written (CERP) and a comprehensive AED program implemented.

## Components of an Evidence-Based AED Program

Although many schools have installed AEDs on their campuses, during an emergency, the AED must be retrieved, applied, and operated by a trained person. In addition, the AED and its supplies must be monitored and maintained. Developing a comprehensive AED program with a CERP ensures that the equipment and personnel will be properly prepared in the event of an emergency. While more research is needed, several studies agree on key details of an AED program. An AED program should include (Boudreaux & Broussard, 2020; Drezner et al., 2019; Kiyohara et al., 2019; Nishiuchi et al., 2018; Saberian et al., 2018):

- AEDS on-site in unlocked, accessible locations.
- All staff are trained to provide CPR and use an AED.
- A CERP with scheduled drills.
- Routine maintenance of AEDs.

The school nurse, as the school campus health advocate, is perfectly positioned to lead the school campus through implementation of a comprehensive AED program. Successful implementation requires collaboration between health services and administration. While the bureaucratic nature of schools creates strengths of consistency, accountability, and stability, it can also challenge innovation, adaptability, and collaboration (Teachers Institute, 2023), which can make districtwide approval of a heart-safe initiative difficult to obtain. While a school board-supported districtwide approach is preferred, an AED program implemented at the school level can allow the nurse to individually address a heart safe initiative for their campus. In situations where each school institutes their own program, goals may be discussed at district nursing meetings to ensure a standardized approach district-wide. School nurses can also access resources from national organizations, like Project ADAM and the American Heart Association, to obtain district buy-in and build a comprehensive plan.

### Addressing Barriers

When implementing a new program, various barriers arise. Financial implications of an AED program must be considered when endeavoring to initiate a program. AED accessibility guidelines recommend having an AED within 3 min of all campus locations (Project ADAM, 2024), which may require schools with larger campuses to purchase and maintain several AEDs. While purchasing AEDs can cost between $1,100 and $3,000 per device, this singular barrier should not deter a school campus from implementing the remaining elements of a comprehensive AED program while funds are being identified for the purchase of AED(s). AED funding options through grants may help to defray costs and working with school or district administrators when budget plans are reviewed can help ensure AEDs are purchased. Luckily, most aspects of a comprehensive AED program can be embedded into preexisting processes without incurring additional costs, including building the operational cost of staff training time into prearranged campus learning days.

In collaboration with the school leadership, the school nurse can implement the AED program by initiating small changes throughout the school year to achieve a comprehensive schoolwide AED program. Hands-only CPR training with inexpensive inflatable practice manikins can be integrated with other health and safety-related trainings that occur at the beginning of the school year. CPR certified staff members can be recruited to assist with campus training and considered for their potential contributive roles during a medical emergency. The nurse can perform monthly AED monitoring, including reviewing AED battery and pad expiration dates, and document through an online form or Excel spreadsheet. Integrating improvements into existing clinic and training processes with consistent reinforcement will achieve sustainability.

## The Cardiac Emergency Response Team and Plan

The cardiac emergency response team (CERT) consists of at least five school personnel who can leave their regular duties immediately and serve as “first responders” to a cardiac emergency on campus (Project ADAM, 2024). The CERT can also be mobilized for any campus medical emergency, not solely cardiac events, thereby serving as a campus-wide medical emergency response team. With medical technology advancements, the number of medically complex students with chronic conditions has increased as well as the risk of medical emergencies occurring at school (Gereige et al., 2022). This is further complicated if there are no licensed health professionals present when a medical emergency occurs and emphasizes the importance of maintaining an established CERT with a practiced CERP (see Table 1). The rising frequency and acuity of campus medical incidents combined with the risk for SCA creates the campus administration buy-in required to collaboratively build a CERT and perform on-site mock drills. The CERT should include:

**Table 1.** Components of a Cardiac Emergency Response Plan

• Develop a CERP. • Establish a CERT with CPR trained staff. • Perform at least one high-quality annual AED drill and practice CERT response, using drill scenarios, checklists, and de-briefs. • Perform annual school staff training, including: ○ established CERP review, ○ CPR and AED use, ○ importance of rapid CPR initiation and AED application, ○ SCA overview. • Designate person responsible for managing AEDs and other emergency equipment, including: ○ documenting AED maintenance, ○ reviewing AED placement and accessibility, ○ ensuring all AED parts are replaced prior to expiration. • Integrate local EMS with school plan. • Document all AED use- with a post de-brief form and hold a postevent debrief meeting. • Review and update CERP and CERT annually.

*Source*. American Heart Association (2023); Project ADAM (2024); Rose et al. (2016).

*Note*. AED = automated external defibrillator; CERP = cardiac emergency response plan; CERT = cardiac emergency response team; CPR = cardiopulmonary resuscitation; EMS = emergency medical services; SCA = sudden cardiac arrest.

- School administrators/principals to direct students, manage the school environment, and contact parents/guardians.
- The school nurse to provide medical care.
- CPR and AED trained school staff to retrieve the AED and provide emergency care, in support of the nurse or in the event the nurse is not present.
- Building Office Personnel to direct emergency medical services (EMS) upon arrival and initiate a school-wide *Hold* via bells and announcements.
- School Liaison Officer (if available) to serve as a liaison between EMS and the school.

Most schools assign an administrator to supervise after school events. Therefore, to ensure there is at least one AED program trained individual at the event, it is recommended that any school employee that will serve in a supervisor role be CPR and AED trained. With the comprehensive AED program in place, the campus administration and nurse can build confidence in the campus staff and students’ families through reassurance of the campus’ ability to respond to medical emergencies.

## Advocating at the State Level

Legal backing with state laws requiring AEDs in schools and CPR and AED training for staff, along with evidence-based implementation guidance, would provide the greatest level of assurance that anyone experiencing a cardiac emergency at school would have the best chance of survival. The *School Nursing Practice Framework* emphasizes the school nurse’s role in advocacy at both the school and legislative levels (NASN, 2024). School nurses can work with parent groups, professional associations, and not-for-profit organizations to combine their voices to advocate for these laws. Ironically, about 40 states require students to learn CPR as a high school graduation requirement, but only about 25 states have any CPR or AED requirement for the school staff employed to keep students safe (Sudden Cardiac Arrest Foundation, n.d.). The state of Texas learned from their tragic loss and signed the Landon Payton Act, Senate Bill 865, on June 20, 2025, requiring all schools receiving TEA funding to develop a comprehensive CERP and implement specified CERP components before the first instructional day of the 2027 to 2028 school year (Landon Payton Act, 2025).

## Call to Action

AED programs with CERPs improve SCA survival outcomes in school-age children and athletes. Many schools lack AED programs with CERPs due to various barriers; overcoming barriers to implementation and achieving widespread implementation is imperative. The school nurse, as the school campus health advocate, is perfectly positioned to lead a school campus through implementation of a comprehensive AED program. Nurses should implement achievable changes incrementally and not delay AED program initiation based on CERP components that may seem daunting or difficult to accomplish. Choose an achievable CERP component and start planning today.
